# Characterization of Ribosomal Frameshifting in Theiler's Murine Encephalomyelitis Virus

**DOI:** 10.1128/JVI.01043-15

**Published:** 2015-06-10

**Authors:** Leanne K. Finch, Roger Ling, Sawsan Napthine, Allan Olspert, Thomas Michiels, Cécile Lardinois, Susanne Bell, Gary Loughran, Ian Brierley, Andrew E. Firth

**Affiliations:** a Division of Virology, Department of Pathology, University of Cambridge, Tennis Court Road, Cambridge, United Kingdom; b de Duve Institute, Université Catholique de Louvain, Brussels, Belgium; c School of Biochemistry and Cell Biology, Western Gateway Building, University College Cork, Cork, Ireland

## Abstract

*Theiler's murine encephalomyelitis virus* (TMEV) is a member of the genus Cardiovirus in the Picornaviridae, a family of positive-sense single-stranded RNA viruses. Previously, we demonstrated that in the related cardiovirus, Encephalomyocarditis virus, a programmed −1 ribosomal frameshift (−1 PRF) occurs at a conserved G_GUU_UUU sequence within the 2B-encoding region of the polyprotein open reading frame (ORF). Here we show that −1 PRF occurs at a similar site during translation of the TMEV genome. In addition, we demonstrate that a predicted 3′ RNA stem-loop structure at a noncanonical spacing downstream of the shift site is required for efficient frameshifting in TMEV and that frameshifting also requires virus infection. Mutating the G_GUU_UUU shift site to inhibit frameshifting results in an attenuated virus with reduced growth kinetics and a small-plaque phenotype. Frameshifting in the virus context was found to be extremely efficient at 74 to 82%, which, to our knowledge, is the highest frameshifting efficiency recorded to date for any virus. We propose that highly efficient −1 PRF in TMEV provides a mechanism to escape the confines of equimolar expression normally inherent in the single-polyprotein expression strategy of picornaviruses.

**IMPORTANCE** Many viruses utilize programmed −1 ribosomal frameshifting (−1 PRF) to produce different protein products at a defined ratio, or to translate overlapping ORFs to increase coding capacity. With few exceptions, −1 PRF occurs on specific “slippery” heptanucleotide sequences and is stimulated by RNA structure beginning 5 to 9 nucleotides (nt) downstream of the slippery site. Here we describe an unusual case of −1 PRF in Theiler's murine encephalomyelitis virus (TMEV) that is extraordinarily efficient (74 to 82% of ribosomes shift into the alternative reading frame) and, in stark contrast to other examples of −1 PRF, is dependent upon a stem-loop structure beginning 14 nt downstream of the slippery site. Furthermore, in TMEV-based reporter constructs in transfected cells, efficient frameshifting is critically dependent upon virus infection. We suggest that TMEV evolved frameshifting as a novel mechanism for removing ribosomes from the message (a “ribosome sink”) to downregulate synthesis of the 3′-encoded replication proteins.

## INTRODUCTION

**FIG 1:**
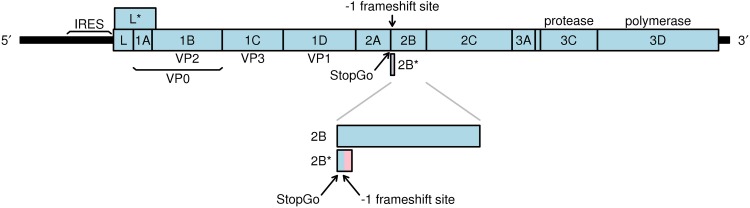
Schematic representation of the TMEV genome. The predicted −1 PRF site is situated at codons 5 and 6 downstream of the junction between the 2A and 2B coding sequences. Frameshift translation would yield a 14-aa transframe protein termed 2B*, whose N terminus would be encoded in the polyprotein frame (blue) and whose C terminus would be encoded in the −1 frame (pink).

The genus Cardiovirus of the family Picornaviridae (a family of positive-sense single-stranded RNA viruses) currently contains two species, Encephalomyocarditis virus (EMCV) and Theilovirus. The latter encompasses a number of divergent viruses, including Theiler's murine encephalomyelitis virus (TMEV), rat theilovirus (RTV), and Saffold virus (SAFV). The approximately 8-kb genome contains a long open reading frame (ORF) that is translated as a polyprotein and subsequently processed by the virus-encoded 3C protease (1–3). Separation of the L-1ABCD-2A and 2BC-3ABCD components, however, occurs cotranslationally via a process termed “StopGo” or “Stop-Carry on” (4–6) (Fig. 1). This unusual proteolysis-independent but ribosome-dependent mechanism is mediated by the amino acid motif D(V/I)ExNPG|P (where the symbol “|” represents the junction between 2A and 2B), together with less conserved but nonetheless functionally important upstream amino acids, which prevent the formation of a peptide bond between Gly and Pro but allow the continuation of translation with up to near-100% efficiency (7–11).

Many RNA viruses contain sequences that stimulate a proportion of ribosomes to undergo a −1 frameshift and to continue translating in an alternative reading frame. Where functionally utilized, this is termed programmed −1 ribosomal frameshifting (−1 PRF). In eukaryotic systems, the stimulatory elements for −1 PRF typically involve a “slippery” heptanucleotide sequence, where the shift in reading frame actually occurs, and a downstream RNA stem-loop or pseudoknot structure. The consensus motif for the shift site sequence is X_XXY_YYZ, where XXX normally represents any three identical nucleotides (though certain exceptions occur, including GGU), YYY represents AAA or UUU, Z represents any nucleotide except G, and underscores separate codons in the initial reading frame (12). During the frameshift, the P- and A-site tRNAs detach from the zero frame codons XXY and YYZ and re-pair in the −1 frame to XXX and YYY. Frameshifting on a suitable shift site is stimulated to high levels (typically to a final −1 PRF efficiency of 5 to 45%, depending on the particular frameshift sequence) by a downstream stimulatory element usually in the form of an RNA pseudoknot or a stem-loop structure, separated from the shift site by a 5- to 9-nucleotide (nt) spacer (13, 14). RNA structures at this distance from the shift site are thought to be located at the mRNA unwinding site of the mRNA entrance channel of the ribosome when the shift site sequence is located within the decoding center (15, 16). Failure to efficiently unwind the RNA structure—perhaps due to the presentation of an unusual topology—is thought to interfere with ribosome progressivity and may also induce tension within the mRNA that leads to uncoupling and subsequent realignment of the codon-anticodon duplexes (15, 17, 18).

Recently, EMCV was shown to utilize −1 PRF at a conserved G_GUU_UUU sequence positioned just downstream of the StopGo site. Frameshifting results in the production of a novel 14-kDa “transframe” protein (termed 2B*) whose N-terminal 11 or 12 amino acids (depending on strain) are encoded within the polyprotein open reading frame (ORF) and whose C-terminal 117 amino acids are encoded by an overlapping ORF in the −1 reading frame (19). In theiloviruses, a G_GUU_UUU sequence is conserved at a similar position in the genome, but a long overlapping ORF in the −1 reading frame is lacking (Fig. 1). If −1 PRF were to occur at this site in these viruses, the resulting transframe protein would be only 14 or 15 amino acids in length.

Here we show that −1 PRF does indeed occur in TMEV and is functionally important for virus propagation. Frameshifting in TMEV is extraordinarily efficient (74 to 82%); to our knowledge, this is the highest frameshifting efficiency reported to date for any virus. We also demonstrate that a 3′ RNA stem-loop structure is involved in frameshift stimulation but acts from a location further downstream of the slippery sequence (14 nt) than is typical (5 to 9 nt). As in EMCV, but in contrast to nearly all other known cases, frameshifting in TMEV depends on virus infection, suggesting the involvement of a frameshift-stimulatory *trans*-acting factor.

## MATERIALS AND METHODS

### Viruses and cell culture.

BHK-21 cells were maintained in Dulbecco's modified Eagle's medium (DMEM) supplemented with 10% fetal bovine serum (FBS), 1 mM l-glutamine, and antibiotics. In virus infections, BHK-21 cells were washed with DMEM containing no serum (SFM) and overlaid with wild-type (WT) TMEV or mutant derivatives at the multiplicities of infection (MOIs) stated below. After a 1-h adsorption period, virus was removed, and cells were overlaid with DMEM supplemented with 2% FBS for various times.

The GDVII strain of TMEV generated from the full-length infectious clone pSK-GDVII was used (a kind gift from the Robert Fujinami lab, University of Utah). The sequence of this clone is identical to GenBank accession no. NC_001366.1 except for three nucleotide differences: G2241A (serine to isoleucine in VP2), A2390G (synonymous change in VP3), and G4437A (lysine to glutamine in 2B). Nucleotide coordinates herein are given with respect to NC_001366.1 (20).

### Recombinant viruses and plasmids.

QuikChange mutagenesis (Agilent Technologies) was performed on a template containing the full-length viral insert to create the full-length recombinant TMEV shift site mutant (SS), stop codon mutant (SCM), and StopGo mutant (LVWT). All constructs were verified by sequencing of the complete virus genome.

V5 and HA-tagged virus variants were created as follows. First, two unique restriction sites (a BsiWI site starting at nt 4166 and a SalI site starting at nt 4536) were introduced (using synonymous changes) into a subclone by site-directed mutagenesis, and the fragment was cloned back into the full-length clone. Two PCR products were then generated using primers overlapping these restriction sites at one end and primers spanning the TMEV sequence and part of the V5 or HA sequence at the other. These fragments fused the first codon following the StopGo junction with the 5′ terminus of the tag coding region and the 3′ terminus of this region with the rest of the 2B/2B* sequence. Different variants were produced to allow the generation of tagged WT, SS, LVWT, and StopGo and shift site mutant (LVSS) clones. The fragments were joined by overlap extension PCR and subcloned back into the modified full-length clone containing the extra restriction sites. The V5 tag, including a glycine-serine linker, was GKPIPNPLLGLDSTGSGSGS, while the HA tag was YPYDVPDYA.

Dual-luciferase frameshift reporter plasmids were prepared by introducing annealed oligonucleotide pairs into XhoI/BglII-digested pIDluc. Plasmid pIDluc is a derivative of the pDluc vector with the mengovirus internal ribosome entry site (IRES) (DQ294633, nt 256 to 770) inserted downstream of the T7 promoter of pDluc such that the second codon of the Renilla luciferase gene is fused to the first seven codons of the mengovirus polyprotein ORF, allowing cap-independent translation.

### *In vitro* transcription and generation of recombinant virus.

Plasmids were linearized with XbaI and transcribed with T7 RNA polymerase (Ambion) for 3 h at 37°C as recommended by the manufacturer. RNA integrity was confirmed by electrophoresis, and RNA was transfected into BHK-21 cells using DMRIE-C (Invitrogen) according to the manufacturer's recommendations. Once cytopathic effect was observed, cells were subjected to three rounds of freeze-thawing, followed by centrifugation for 5 min at 5,000 rpm to pellet cell debris. Virus-containing supernatant was titrated and stored at −80°C.

### Plaque assays.

BHK-21 cells at ∼70 to 80% confluence in 6-well plates were infected with 10-fold dilutions of virus for 1 h at 37°C. Cells were overlaid with DMEM supplemented with carboxymethyl cellulose (CMC; ∼0.35% each of low- and high-viscosity CMC) and 2% FBS and incubated at 37°C for 48 h. After incubation, cells were fixed using formal saline and stained using 0.1% toluidine blue.

### One-step growth curves.

BHK-21 cells were infected with WT, SS, SCM or LVWT TMEV at an MOI of 10 and incubated at 37°C for 1 h. Cells were washed twice in ice-cold phosphate-buffered saline (PBS) and overlaid with 2% DMEM. Virus was harvested at time points indicated below, and the titers were determined using a plaque assay.

### Competition assays.

BHK-21 cells in six-well plates were infected with either WT and SS or WT and SCM at an MOI of 0.1. After adsorption, cells were washed with PBS and overlaid with 2% DMEM (2 ml) and incubated for 24 h, after which virus was harvested (passage 1) and 250 μl was used to reinfect fresh BHK-21 cells. This was repeated five times. RNA was extracted from virus harvested at each passage using TRIzol-LS (Sigma) according to the manufacturer's instructions. A total of 1,500 ng of RNA was reverse transcribed using avian myeloblastosis virus (AMV) reverse transcriptase (Promega) for 1 h at 42°C. The cDNA generated was used as the template for PCR amplification of a region encompassing the frameshift site using specifically designed primers. PCR products were purified using the Promega PCR purification kit and subsequently sequenced.

### Metabolic labeling.

BHK-21 cells at 90 to 100% confluence in 24-well plates were infected with WT, SS, SCM, or LVWT TMEV at an MOI of 10. After 60 min adsorption at 37°C, virus inocula were removed by aspiration and replaced with 2% DMEM, and the cells were incubated at 37°C for 6 h. Cells were subsequently washed once with methionine-free DMEM and incubated in 1 ml of methionine-free DMEM for 1 h. Cells were labeled with [^35^S]methionine (200 μCi/ml) at 37°C for 1 h. Cells were scraped into 0.4 ml ice-cold PBS and spun at 5,000 rpm for 5 min. Cell pellets were washed with ice-cold PBS, resuspended in 70 μl Laemmli's sample buffer, heated at 95°C for 5 min, and analyzed by SDS-PAGE (6 to 15%). After electrophoresis, gels were fixed, dried, and subjected to phosphorimaging. Frameshifting efficiencies were estimated from band intensities quantified using the software ImageQuant TL, as described in Results.

### Antibodies.

The antibody (Ab) against TMEV protein VP1 (mouse monoclonal) has been described previously (21). Rat monoclonal anti-tubulin Ab, mouse monoclonal anti-HA Ab, and agarose-conjugated V5 and HA antibodies used for the immunoprecipitation of tagged viral proteins were from Sigma. Mouse monoclonal anti-V5 Ab was from Life Technologies, and IRDye-conjugated secondary antibodies used for immunoblotting were from Li-Cor.

### Immunoblotting.

BHK-21 cells were infected with TMEV WT or mutants thereof at an MOI of 10. Infected cells were lysed directly in Laemmli's sample buffer and heated to 95°C for 5 min. Proteins were separated on gels as indicated in the figure legends and transferred to nitrocellulose membranes. These were blocked for 30 to 60 min with 5% powdered milk (Marvel) in PBS containing 0.1% Tween 20 (PBST) and probed at 4°C overnight with primary antibody. Membranes were washed in PBST prior to incubation in the dark with an IRDye-conjugated secondary antibody in PBST. Blots were scanned using an Odyssey infrared imaging system (Li-Cor).

### Mass spectrometry.

Infected BHK-21 cells from a 25-cm^2^ flask were washed in cold PBS and lysed in radioimmunoprecipitation assay (RIPA) buffer (50 mM Tris HCl [pH 8], 150 mM sodium chloride, 1% NP-40 substitute, 0.5% sodium deoxycholate, 0.1% SDS) plus protease inhibitors and Benzonase (Merck) on ice for 20 min. Cell debris was removed by centrifugation at 21,000 × *g* at 4°C for 10 min. Cell lysates were incubated with 50 μl of protein A Sepharose in RIPA buffer and 0.5 μl of an irrelevant goat antibody for 1 h at 4°C with gentle mixing. Lysates were centrifuged through SigmaPrep columns at 8,200 × *g* at 4°C for 1 min, and the supernatant was incubated overnight with 200 μl of anti-V5 conjugated agarose beads (Abcam) on an end-over-end mixer at 4°C. Beads were washed three times in a high-salt wash (0.5 M LiCl, 0.1 M Tris-HCl [pH 8.5]) before resuspension in Laemmli's sample buffer and boiling for 7 min to remove protein from the beads. Proteins were separated by electrophoresis on a 12% bis-Tris urea gel run in morpholineethanesulfonic acid (MES) buffer (Invitrogen) at 30 to 40 mA for optimal resolution. The gel was washed and stained using colloidal Coomassie blue, and bands were excised for mass spectrometry.

Coomassie blue-stained products were excised from the gel and subjected to in-gel trypsin digestion. Peptides were extracted and analyzed by liquid chromatography-tandem mass spectrometry (LC-MS/MS) using an Agilent 1200 series nanoflow system (Agilent Technologies) connected to a LTQ Orbitrap mass spectrometer (Thermo Electron) equipped with a nanoelectrospray ion source (Proxeon). Fragment MS/MS spectra were searched with the Mascot 2.3 search engine (Matrix Science) against a protein sequence database composed of expected viral target sequences and common contaminant proteins such as trypsin and keratins. Search parameters included a 5-ppm precursor mass tolerance and 0.6-Da MS/MS mass tolerance, three missed trypsin cleavages, and trypsin cleavage before proline plus a number of variable modifications, such as oxidation (M), oxidation (HW), phospho (ST), and phospho (Y). For the figures, spectra were autoannotated with xiSPEC (http://spectrumviewer.org).

### Dual-luciferase assays.

BHK-21 cells were transfected in triplicate with the relevant plasmid using Lipofectamine 2000 reagent (Invitrogen) in a 24-well plate. Transfected cells were incubated at 37°C for 18 h prior to infection with WT TMEV at an MOI of 10. Luciferase activities were determined using the dual-luciferase Stop & Glo reporter assay system (Promega) at 7 h postinfection (p.i.). Transfected cells were washed once with PBS and lysed in 100 μl of 1× passive lysis buffer, and light emission was measured following injection of 50 μl of either Renilla or firefly luciferase substrate. Firefly luciferase activity was calculated relative to the activity of Renilla luciferase, and frameshifting efficiencies were determined by comparing the ratio of firefly to Renilla enzymatic activities in parallel cell cultures transfected with either the test construct or an in-frame control (IFC), as described previously (24).

## RESULTS

### Mutating the predicted frameshift site attenuates virus growth.

**FIG 2:**
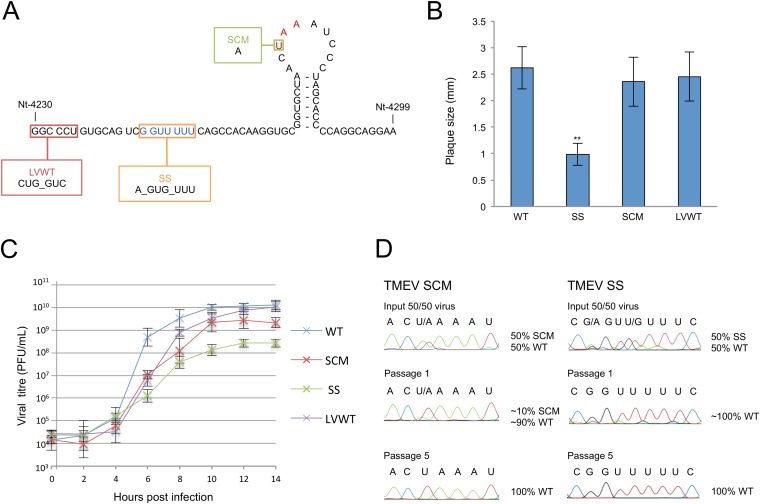
Analysis of TMEV mutants. (A) Schematic representation of mutations. SS, shift site mutant; SCM, 2B* extension mutant; LVWT, StopGo mutant. The shift site sequence is in blue, and the 2B* stop codon is in red. (B) Mean plaque sizes for TMEV WT and mutant viruses. Mean plaque sizes are the averages for 100 representative plaques; error bars indicate standard deviations. **, SS is significantly different from WT (two-tailed *t* test, *P* < 0.001). (C) One-step growth curves. BHK-21 cells were infected with WT, SS, SCM, or LVWT viruses at an MOI of 10 and harvested at the indicated time points. Titers were measured by plaque assay. At least two biological repeats were performed for each virus; error bars indicate standard deviations. (D) Competition assay. BHK-21 cells were infected with a mixture of an MOI of 0.1 of either SS plus WT or SCM plus WT viruses. At 24 h p.i., virus was harvested and used to reinfect BHK-21 cells. Passaging was repeated five times. RNA was extracted from passages 0, 1, and 5, and a region of ∼1,000 nt encompassing the mutated region was sequenced.

In the GDVII strain of TMEV, the mass of the predicted 14-amino-acid (aa) transframe fusion protein that would arise from −1 PRF is only 1.4 kDa. Products of such small size are inherently difficult to detect by SDS-PAGE and/or Western blotting. Therefore, we decided to first seek genetic evidence for the functional importance of the predicted frameshift site in TMEV through mutagenesis of an infectious clone. Three mutants were generated (Fig. 2A): (i) G_GUU_UUU to **A**_GU**G**_UUU (mutations are in bold) at nt 4244 to 4250 to prevent frameshifting at the predicted shift site (SS); (ii) ACU_AAA to AC**A**_AAA at nt 4272 to 4277 to remove the UAA stop codon of the predicted 2B* product, thus extending it by a further 21 aa in length (SCM); and (iii) GGC_CCU to **CUG_GUC** at nt 4230 to 4235 to inhibit StopGo by introducing GP-to-LV amino acid changes at the junction between 2A and 2B (LVWT). This mutation was shown previously to inhibit StopGo from occurring, thereby fusing the 2A and 2B (or 2B*) proteins together, and this mutant was constructed to assess the relative importance of StopGo and frameshifting for TMEV replication and to assess whether StopGo affects frameshifting. The SS and SCM mutations do not change the polyprotein amino acid sequence.

RNA transcribed from the WT, SS, SCM, and LVWT molecular clones was transfected into BHK-21 cells to generate virus stocks which were analyzed by plaque assay. While the StopGo mutant (LVWT) and 2B* extension mutant (SCM) produced plaque sizes similar to those of the WT, the shift site mutant (SS) gave significantly smaller plaques (Fig. 2B). The growth phenotypes of WT, SS, SCM, and LVWT viruses were analyzed further using one-step growth curve analysis (Fig. 2C). As in the plaque assays, SS exhibited a marked defect in growth kinetics, with peak titers 1.5 log lower than those of the WT. SCM exhibited a slight attenuation in replication, reaching peak titers around half a log lower than those observed for WT. In agreement with results reported by Loughran and colleagues (22), the LVWT growth curve closely mirrored that of the WT, with only a slight lag in replication kinetics.

To further investigate fitness of the SCM and SS mutants, a competition assay was performed, whereby a mixture of equal amounts of WT and mutant virus was used to infect BHK-21 cells and the sequences of the resulting progeny were determined following RNA extraction. At various passage numbers, we assessed whether and how quickly the WT virus was able to outcompete either mutant. Consistent with the single-cycle growth curves described above, WT virus was able to outcompete SS within one passage (approximately two full WT replication cycles), while for SCM, after one passage, the ratio of SCM to WT RNA was approximately 1:10. At later passages, the SCM sequence disappeared completely, leaving only the WT sequence (Fig. 2D). Together, these results provide genetic evidence for the functional importance of the predicted frameshift site in TMEV.

### Frameshifting in the viral context is highly efficient.

If frameshifting does indeed occur in TMEV, then, since ribosomes that frameshift would terminate and dissociate from the message at the 2B* stop codon (Fig. 1), there should be fewer ribosomes synthesizing polyprotein products encoded downstream of the frameshift site (2BC-3ABCD) than polyprotein products encoded upstream of the frameshift site (L-1ABCD-2A). To investigate this, we used metabolic labeling to quantify viral protein production. In TMEV, the frameshifting efficiency cannot be robustly estimated simply by analyzing the ratio of upstream and downstream products for WT virus, due to the presence of intermediate polyprotein processing products, as well as potential differences in protein turnover rates. However, by normalizing WT protein levels to the corresponding levels for the SS mutant—in which proteins encoded upstream and downstream of the shift site are expected to be produced in equimolar amounts—we can at least partly correct for these confounding factors.

**FIG 3:**
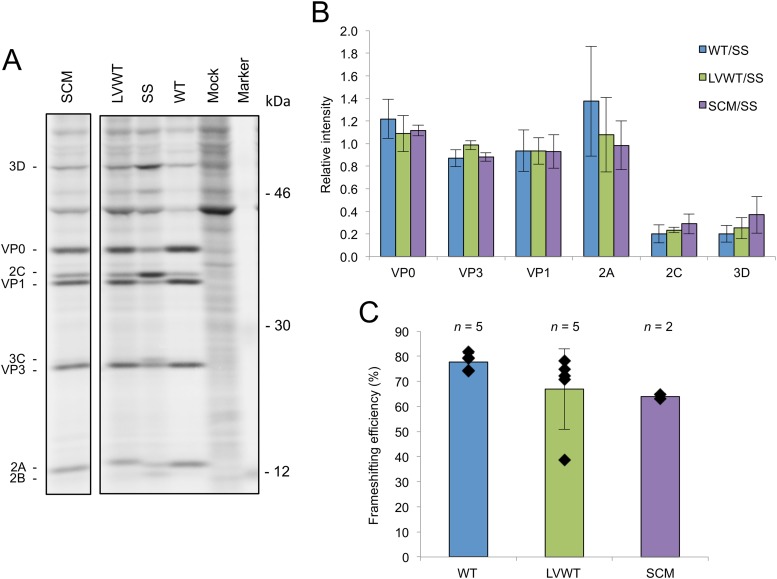
Analysis of frameshifting in the viral context. (A) Radiolabeled TMEV translation products. BHK-21 cells were infected with either WT, SCM, SS, or LVWT viruses at an MOI of 8 or mock infected. Cells were labeled from 6 to 7 h p.i. and harvested at 7 h p.i., and proteins were separated by SDS-PAGE. Note that the more slowly migrating 2A band for LVWT may contain both 2A-2B* and a 2A-2B cleavage product. All samples were run on the same gel; an irrelevant lane has been excised. (B) Relative amounts of TMEV proteins. Individual band intensities for WT, LVWT, and SCM were normalized first by methionine content, then by the means of these values for VP0, VP1, and VP3 (to control for lane loading), and then by the corresponding similarly normalized band for SS. Each bar represents the mean (± standard deviation) from all biological repeats in which the corresponding band could be resolved and quantified (see the text). (C) Frameshifting efficiency. The intensity in each of the VP0, VP3, VP1, and 2C bands for WT, LVWT, and SCM viruses was normalized first by methionine content, then by the means of these values for VP0, VP1, and VP3 (to control for lane loading), and then by the corresponding similarly normalized values for SS. Then the value for 2C (downstream product) was divided by the average of the values for VP0, VP3, and VP1 (upstream products). Subtracting this value from 1 and multiplying the result by 100 gives the percent frameshifting efficiency. Each bar represents the mean value (± standard deviation) from five, five, and two biological repeats for WT, LVWT, and SCM viruses, respectively.

[^35^S]methionine-labeled proteins from infected cell lysates were separated by electrophoresis and quantified by phosphorimaging. The expression levels of products encoded downstream of the predicted shift site were observed to be much lower, relative to the upstream products, for WT virus than for the SS mutant (Fig. 3A), indicating that a substantial proportion of ribosomes leave the polyprotein frame in the vicinity of the frameshift site and that this departure is mediated by the sequence of the predicted frameshift site. This is most obvious when the intensities of the 2C and VP1 proteins are compared. The intensity of each band depends on the number of methionines present and the abundance of the protein. There is clearly much more of the downstream product 2C relative to VP1 in the SS sample than in the WT, LVWT, and SCM samples.

The relative abundances of individual TMEV proteins were calculated by normalizing the intensity of each band first by the number of methionines in the corresponding product, then by the means of these values for VP0, VP1, and VP3 (to control for lane loading), and finally by the corresponding similarly normalized band for the SS mutant (Fig. 3B). This highlights the substantial fall in relative protein levels between the upstream products (VP0, VP3, VP1, and 2A) and the downstream products (2C and 3D). Due to the relatively high background and low intensity of the 2B and 3C bands, it was not possible to reliably estimate the levels of these proteins for the WT and LVWT viruses, although their relative expression levels are clearly much lower than for the SS mutant (Fig. 3A).

In order to calculate frameshifting efficiencies, the intensity of each of the VP0, VP3, VP1, and 2C bands from WT, LVWT, and SCM was normalized first by methionine content, then by the mean of these values for VP0, VP1, and VP3 (to control for lane loading), and then by the corresponding similarly normalized values for the SS mutant as described above. Then, the value for 2C (downstream product) was divided by the average of the values for VP0, VP3, and VP1 (upstream products). (2A and 3D were excluded, as these products were not always clearly resolved and quantifiable.) This provides an estimate of the fraction of ribosomes that avoid a −1 PRF. Subtracting this value from 1 and multiplying the result by 100 gives the percent frameshifting efficiency (Fig. 3C). Using this method, the frameshifting efficiency for WT virus was estimated as 74 to 82% (95% confidence interval; *n* = 5) (Fig. 3C). With the exception of one outlier, LVWT behaved similarly to the WT virus (*n* = 5) (Fig. 3C). SCM had a frameshifting efficiency of 53 to 75% (95% confidence interval; *n* = 2) which was significantly lower than the WT frameshifting efficiency (*P* = 0.003, 2-tailed pooled variance *t* test), indicating that the SCM mutation may have partly interfered with a frameshift-stimulating element. It should be noted that this calculation assumes that ribosomes either frameshift or continue translating in the polyprotein frame; however, it is also possible that a proportion of ribosomes prematurely terminate at the frameshift site (23).

### Mass-spectrometric confirmation of 2B* expression and the site of frameshifting.

**FIG 4:**
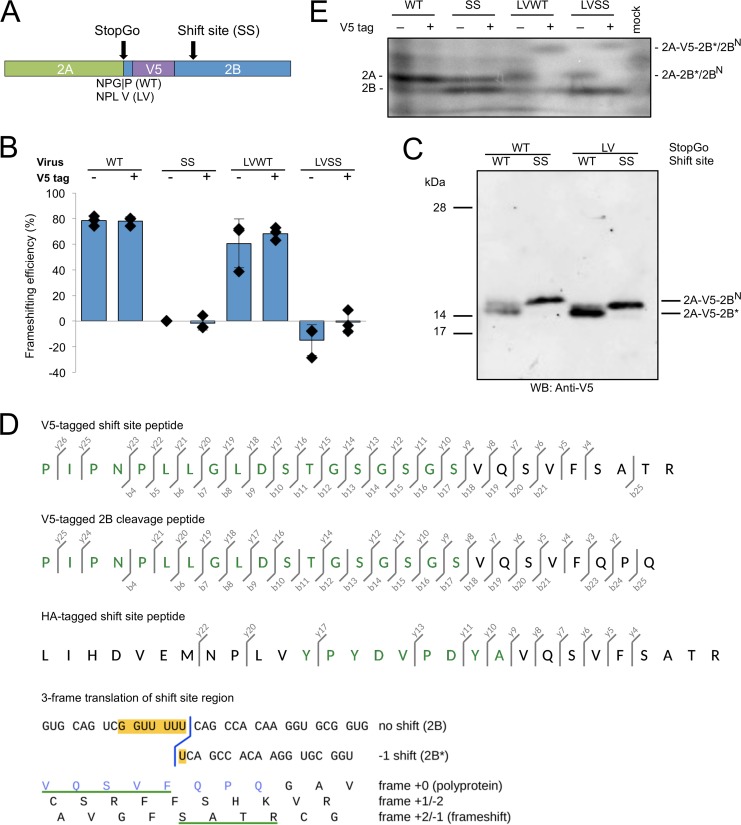
Characterization of tagged viruses. (A) Schematic representation of the tagged viruses. A V5 tag was inserted just after the first proline of 2B in the WT sequence (after valine in the GP-to-LV StopGo-mutated viruses), to tag products containing 2B or 2B*. (B) Frameshifting efficiencies of tagged and untagged viruses. BHK-21 cells were infected with WT, SS, LVWT, or LVSS viruses or their tagged equivalents, and frameshifting efficiencies were calculated from radiolabeled products as described in Fig. 3C. All viruses were normalized by SS; thus, the frameshifting efficiency for SS is zero by definition. Negative frameshifting efficiencies are likely an artifact of measurement errors and/or biological variability. Each bar represents the mean value (± standard deviation) from three biological repeats (the three untagged WT and LVWT data points are also used in Fig. 3C). (C) Western blot of virus-infected cell lysates. BHK-21 cells were infected with V5-tagged viruses at MOI of 1 for LVSS, 5 for SS, and 10 for WT and LVWT, and lysates were prepared when cytopathic effect was extensive. Fivefold-smaller amounts of the LVWT and LVSS samples were loaded to give band intensities similar to those of the WT and SS samples, where only proteins generated by StopGo failure are detected. The proteins with the GP-to-LV mutation may migrate slightly faster than the wild-type ones. Samples were run on bis-Tris gels with 6 M urea and MOPS buffer, which results in altered mobility of the prestained 14-kDa and 17-kDa markers. (D) Mass spectrometric analysis of tagged products. Lysates from BHK-21 cells infected with V5- or HA-tagged TMEV LVWT were immunoprecipitated with V5 or HA antibodies, respectively, and immunoprecipitates were separated by SDS-PAGE. Products migrating at the expected size for 2A-tag-2B* were subjected to in-gel trypsin digest, and peptides were analyzed by LC-MS/MS. Fragmentation ions are shown for the shift site peptide derived from V5-tagged 2A-2B* (top), a peptide consistent with 3C-Pro cleavage of tagged 2A-2B at the conserved Q|G encoded just downstream of the frameshift site (middle), and the shift site peptide derived from HA-tagged 2A-2B* (bottom). Amino acids derived from the V5 or HA tag are in green. The b- and y-series ions correspond to N- and C-terminal fragments. See Fig. S1 in the supplemental material for the fragmentation spectra. Below, the nucleotide sequence in the vicinity of the shift site G_GUU_UUU is shown, with conceptual amino acid translations in all three reading frames. The C-terminal end of the frameshift tryptic peptide is underlined in green, and the C-terminal end of the 2B cleavage peptide is in blue. (E) Low-molecular-mass radiolabeled TMEV translation products. BHK-21 cells were infected with WT, SS, LVWT, or LVSS viruses or their tagged equivalents at an MOI of 10 or mock infected. Cells were labeled from 8 to 9 h p.i. and harvested at 9 h p.i., and proteins were separated by SDS-PAGE.

To confirm −1 PRF at the predicted frameshift site and translation of the predicted 2B* peptide, mass spectrometry was utilized. Purification of WT 2B* proved to be impracticable given its small size (1.4 kDa) and poor predicted antigenicity. Therefore, we generated viruses expressing V5-tagged 2B*. Sequences encoding a V5 tag and linker were inserted just after the last codon of the StopGo sequence (proline in the WT virus and valine in the LV mutants) in the WT, SS, LVWT, and LVSS (a poorly growing mutant in which both StopGo and the shift site were disabled) viruses (Fig. 4A). In this location, V5 is expected to tag both 2B and 2B* near their N termini. Frameshifting efficiencies were estimated from radiolabeled products as described above and were found to be similar to those of the respective untagged viruses (Fig. 4B). These data indicate that the V5-tagged viruses mimic their untagged counterparts during virus replication in cell culture.

Following this initial characterization, BHK-21 cells were infected with V5-tagged WT, SS, LVWT, or LVSS viruses and lysed when cytopathic effect was extensive. Proteins were separated by SDS-PAGE, and V5-tagged products were detected by Western blotting (Fig. 4C). For V5-tagged WT virus, no product was observed migrating at a size compatible with that expected for V5-tagged 2B* (3.3 kDa), suggesting that this product may be rapidly degraded (data not shown). The predicted size of the V5-tagged 2A-2B* fusion that would be produced when StopGo is inhibited in LVWT virus is 18.8 kDa. A doublet (possible explanation below) migrating at a position consistent with this size was observed for both the V5-tagged WT and LVWT viruses, whereas for the V5-tagged shift site mutant viruses (SS and LVSS), only a single band was observed to migrate at this position. We hypothesized that the additional band seen in WT and LVWT, but not in the shift site mutants, represented the frameshift product 2A-V5-2B* (18.8 kDa) (produced at a low level in V5-tagged WT virus only when StopGo separation fails; see below).

Next, we performed an anti-V5 immunoprecipitation using lysate from cells infected with V5-tagged LVWT. Immunoprecipitates were separated by SDS-PAGE and stained with colloidal Coomassie blue. Again, a doublet was observed migrating at the appropriate size. Both bands were excised and digested with trypsin, and the resulting peptides were analyzed by liquid chromatography-tandem mass spectrometry (LC-MS/MS). Peptides covering 98% or 96% of the predicted 2A-V5-2B* fusion were identified from each band in the doublet (although some of these peptides may have arisen from comigrating nonframeshift products; see below). Importantly, however, the peptide encoded by the shift site sequence was identified, thus confirming both the site and the direction (−1) of the frameshift (Fig. 4D; also, see Fig. S1A in the supplemental material).

Peptides were also mapped to a portion of the 2B protein. Near the N-terminal end of 2B, there is a potential 3C-Pro cleavage site, QG, that is conserved in theiloviruses and EMCV (19). When StopGo fails (WT) or is inhibited (LV), cleavage at this site would produce a V5-tagged product comprising 2A fused to V5 and the N-terminal 9 aa of 2B. This product (2A-V5-2B^N^) has a predicted mass of 18.4 kDa and would migrate close to 2A-V5-2B*. Peptides confirming usage of this cleavage site were observed during mass spectrometry of both bands (Fig. 4D; also, see Fig. S1B in the supplemental material). Thus, the doublet seen in the Western blot for V5-tagged LVWT may comprise both 2A-V5-2B* and 2A-V5-2B^N^, with the detection of both products in both bands simply indicating that the doublet was not resolved well enough to avoid contamination between the two gel slices. This doublet was also observed for V5-tagged WT virus, and this likely results from a proportion of ribosomes reading through the StopGo cassette without cotranslational separation occurring, leaving a proportion of 2A fused to V5 and 2B* or 2B^N^.

A sequence encoding an HA tag was also inserted into the LVWT backbone, and anti-HA immunoprecipitation was used to purify 2A-HA-2B* for LC-MS/MS analysis. The sequence data obtained were consistent with that of the V5-tagged viruses, with high coverage of the 2A-HA-2B* product and recovery of the shift site peptide, again verifying the site and direction of frameshifting (Fig. 4D; also, see Fig. S1C in the supplemental material). These experiments confirm that −1 PRF does indeed occur on the G_GUU_UUU motif during translation of the TMEV genome.

These findings also help explain the migration patterns observed for the low-molecular-mass radiolabeled products of the eight viruses (Fig. 4E). In the untagged StopGo mutant viruses, a product migrating slightly more slowly than 2A may represent comigrating 2A-2B* and 2A-2B^N^ in LVWT and 2A-2B^N^ alone in LVSS. In the tagged StopGo mutant viruses, this was replaced with a still more slowly migrating band, consistent with 2A-V5-2B* or 2A-V5-2B^N^ (the three prolines in the V5 tag may explain their aberrant migration relative to 2A and 2A-2B*/2A-2B^N^). In contrast, the band presumed to correspond to 2B (note that the other downstream low-mass viral products, 3A and 3AB, lack methionines) did not shift between untagged and tagged viruses (e.g., SS) (Fig. 4E), nor between wild-type and mutant StopGo viruses (e.g., cf. SS with LV-SS in Fig. 4E), suggesting that this band actually represents N-terminally cleaved 2B.

### Frameshifting is stimulated by virus infection and requires a 3′ stem-loop structure.

Previously, frameshifting in the related EMCV was shown to depend upon virus infection and on at least 50 nt of the 3′ sequence (19). The EMCV 3′ sequence is predicted to form a stem-loop at an unusual distance from the shift site. However, the role of the predicted structure in EMCV frameshifting remains uncertain, as all mutations tested in a reporter system—even ones predicted to restore the stem-loop structure but with an altered sequence—inhibited frameshifting (19). A stem-loop with similar spacing (14 nt) is also predicted in TMEV (Fig. 2A). To investigate the important elements for TMEV frameshifting, we cloned the relevant region into a dual-luciferase reporter construct, pIDluc, which is a modified version of pDluc (24, 25). As picornavirus infection results in host translational shutoff, pDluc was adapted to contain the EMCV IRES to allow cap-independent translation during viral infection. The TMEV WT shift site sequence (G_GUU_UUU) or mutated shift site sequence (SS; A_GUG_UUU), together with 6 nt of upstream sequence and 92 nt of downstream sequence (including the predicted stem-loop structure), was cloned between the Renilla and firefly luciferase ORFs in pIDluc such that frameshifting is required for expression of the firefly ORF. Two additional changes were introduced: a U-to-C mutation (removing the −1 frame stop codon) was introduced into all plasmids to allow −1 frame translation to continue into the downstream firefly luciferase, and a CAA-to-UAA mutation was introduced to generate a stop codon in the zero frame at the third codon after the shift site. The latter mutation was introduced as preliminary experiments indicated that, without it, the extended C-terminal tail that is appended to the Renilla luciferase when frameshifting failed to occur was inhibiting its enzymatic activity. An in-frame control (IFC) in which the firefly ORF was placed in the same frame as the Renilla ORF by inserting an extra U at the end of the G_GUU_UUU shift site was also constructed.

**FIG 5:**
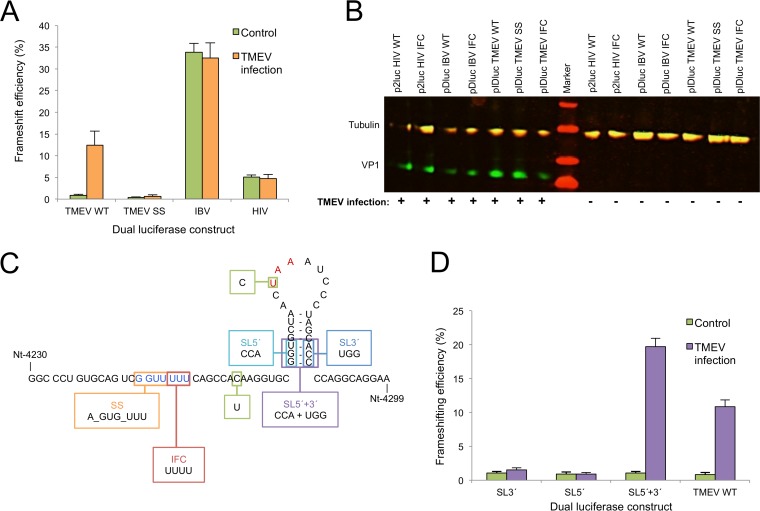
Analysis of frameshift stimulators. (A) Frameshifting efficiencies measured using dual-luciferase constructs. BHK-21 cells were transfected with frameshift reporter constructs and 18 h later were either infected with WT virus at an MOI of 10 or mock infected. Lysates were harvested at 7 h p.i. and assayed for Renilla and firefly luciferase activity. Frameshift efficiencies were determined by comparing luciferase activities to an in-frame control (IFC) construct. Mean values and standard deviations are shown, each based on nine separate transfections. (B) Western blot verifying infection of infected samples. Aliquots of each of the cell lysates were separated on a 10 to 20% Tris-Tricine gradient gel and probed with rat monoclonal anti-tubulin (red, IRDye 700-labeled secondary) and mouse monoclonal anti-VP1 (TMEV capsid protein) (green, IRDye 800-labeled secondary) antibodies. Note that the rat monoclonal primary cross-reacts with both the secondary antibodies. (C) Schematic representation of the fragments cloned into the pIDluc vector. All constructs contain the U-to-C mutation removing the −1 frame UAA stop codon (red) to allow expression of the downstream luciferase and the C-to-U mutation to introduce a zero-frame UAA stop codon just 3′ of the shift site (see the text). pIDluc IFC contains an extra U in the G_GUU_UUU shift site sequence (blue). (D) Frameshifting efficiencies of dual-luciferase constructs containing stem-loop mutants. See the description of panel A for details. Mean values and standard deviations are shown, each based on nine separate transfections.

Plasmids pIDluc SS, pIDluc WT, and pIDluc IFC were transfected into BHK-21 cells 18 h prior to either mock infection or infection with WT virus at an MOI of 10. Frameshifting efficiencies were measured to be <1% for pIDluc WT in mock-infected cells but ∼12 to 13% in TMEV-infected cells, showing that, as in EMCV, frameshifting on the TMEV frameshift sequence is dependent on virus infection (Fig. 5A). As expected, pIDluc SS failed to support frameshifting either with or without virus infection. To test whether frameshift stimulation by TMEV infection is restricted to the TMEV frameshift signal, two other dual-luciferase constructs harboring unrelated frameshift signals from infectious bronchitis coronavirus (pDluc IBV) and human immunodeficiency virus type 1 (p2luc HIV) (26), and their corresponding IFCs were tested alongside the TMEV constructs. In these constructs, frameshifting occurred in mock-infected cells at levels consistent with previous work (26), and the frameshifting efficiencies were not increased by TMEV infection (Fig. 5A and B).

Canonical eukaryotic −1 PRF is stimulated by a stable 3′ RNA secondary structure separated from the shift site by a 5- to 9-nt spacer sequence. However, the predicted stem-loop in EMCV and TMEV is separated from the shift site by 13 or 14 nt (19). In order to evaluate whether the predicted stem-loop is involved in the stimulation of frameshifting in TMEV, three mutants were generated (Fig. 5C): (i) pIDluc SL5′, where the first three bases of the 5′ half of the stem were mutated (5′-GGU-3′ to 5′-CCA-3′); (ii) pIDluc SL3′, where the last three bases of the 3′ half of the stem were mutated (5′-ACC-3′ to 5′-UGG-3′); and (iii) pIDluc SL5′+3′, where both sets of mutations were combined so as to restore the predicted structure but with reversed base pairings. For each mutant, a corresponding IFC was constructed. Frameshifting efficiencies for both pIDluc SL3′ and pIDluc SL5′ were found to be negligible even with TMEV infection (Fig. 5D). In contrast, the frameshifting efficiency for the restoration mutant (pIDluc SL5′+3′) was ∼19% when stimulated by TMEV infection, similar to that for pIDluc WT. This strongly indicates that the predicted 3′ RNA stem-loop structure does indeed form and plays a critical role in the stimulation of frameshifting in TMEV.

## DISCUSSION

We have demonstrated that TMEV utilizes −1 PRF at a conserved G_GUU_UUU sequence in the 2B-encoding region of the polyprotein ORF. A virus (the SS mutant) with the predicted frameshift site disabled by mutations synonymous in the polyprotein frame exhibited a small-plaque phenotype and attenuated growth kinetics compared to WT virus. Moreover, using a V5-tagged StopGo mutant virus, a product of the expected size for frameshift translation was detected by anti-V5 Western blot, and the site and direction of frameshifting confirmed by mass spectrometry of this product. Analysis of the ratio of structural to nonstructural proteins using metabolic labeling, comparing WT virus with the SS mutant revealed that frameshifting efficiency in the viral context is exceptionally high, at 74 to 82%.

When the TMEV frameshift cassette was cloned into a dual-luciferase reporter, frameshifting was found to be dependent upon viral coinfection, indicating that a virus-stimulated *trans*-acting factor may be required for efficient frameshifting. Frameshift stimulation also involves a downstream RNA stem-loop structure at a noncanonical spacing (14 nt) from the frameshift site. While canonical −1 PRF-stimulatory RNA structures (5- to 9-nt spacing) are expected to be positioned partly within the mRNA entrance channel at the onset of frameshifting, the cardiovirus stem-loop structure will be located close to the leading edge of the ribosome when the P and A sites are positioned on the shift site sequence. One possible explanation for the observations may be that a virus protein, or virus-stimulated host protein, binds to the stem-loop structure and that this RNA-protein complex is able to mimic a canonical −1 PRF-stimulatory RNA structure at a different spacing or otherwise interact with the ribosome to promote −1 PRF. Recent work on *Porcine reproductive and respiratory syndrome virus* (family Arteriviridae) has demonstrated that in at least one other case of ribosomal frameshifting, the 3′ stimulator involves mRNA-protein interactions (27).

Frameshifting efficiencies in the virus context were estimated from polyprotein processing products quantified by metabolic labeling. Although we cannot rule out the possibility that the kinetics of polyprotein processing may be modified in the mutants, the very high efficiency of the TMEV frameshifting signal is consistent with recent metabolic labeling and ribosome profiling data for the cardiovirus EMCV, where frameshifting was found to be 50 to 70% efficient (R. Ling , J. D. Jones, I. Brierley, and A. E. Firth, unpublished data). Why frameshifting is noticeably less efficient in the context of the dual-luciferase reporter system (although still TMEV infection dependent) is uncertain, although similar observations have been reported for EMCV (7% in pDluc [20]). One possibility is that distal frameshift-stimulatory elements exist in the virus genome that are not present in the dual-luciferase reporter vector. A more likely explanation, however, is that the reduced frameshifting efficiency is a consequence of a relative buildup of the nonframeshift product (Renilla luciferase) in cells prior to the onset of virus-stimulated frameshifting. A reduced frameshifting efficiency would perhaps also be observed if the dual-luciferase reporter mRNA and “virus transactivator” were not appropriately localized in the cell or if the reporter was present at an inappropriate molar ratio.

Frameshifting at the same genomic location was previously demonstrated in the related cardiovirus, EMCV (19). However, in EMCV, frameshifting results in the production of a much larger transframe 2B* protein (128 or 129 aa, depending on the isolate) than in TMEV (14 aa). In EMCV, a mutation (PTC2) that truncated 2B*, but that was not expected to interfere with frameshifting, produced an intermediate phenotype, suggesting that both the 2B* protein and frameshifting *per se* are functionally important (19). In TMEV, frameshifting is critical for efficient viral growth in cell culture, but it is unclear whether the 14-aa 2B* has a function in its own right or whether it is simply the by-product of a functionally important frameshift. Frameshifting in TMEV diverts 74 to 82% of ribosomes out of the polyprotein reading frame, thus greatly reducing expression of the 3′-encoded replication proteins relative to the 5′-encoded structural proteins. This, in itself, may provide a selective advantage, as the structural proteins are required in much greater quantities than the replication proteins. Although the mutant with 2B* extended (the SCM mutant) exhibited a slight defect in growth kinetics compared to WT, this could be due to a reduction in frameshifting efficiency since the mutation is within the frameshift-stimulatory stem-loop structure and radiolabeling indicated that this mutant had a decreased frameshifting efficiency (Fig. 3C). At this time, however, a functional role for TMEV 2B* cannot be ruled out.

The StopGo process that cotranslationally separates the polyprotein between 2A and 2B is conserved between TMEV and EMCV and is essential for efficient replication in EMCV (8). However, consistent with previous results (22), our data show that StopGo in TMEV is not crucial for efficient viral growth in cell culture, as judged by the similar growth kinetics and plaque phenotypes exhibited by LVWT and WT viruses. In addition, inhibiting StopGo had no or only a slight detrimental effect on the efficiency of ribosomal frameshifting (Fig. 3C). It has been suggested that the apparent redundancy of StopGo in TMEV may partly stem from the presence of a potential 3C-Pro cleavage site within 2B (QG, encoded just downstream from the shift site; also conserved in EMCV) (22). Cleavage at QG in a StopGo mutant would yield 2A fused to the N-terminal 9 amino acids of 2B (2A-2B^N^) and a separate N-terminally truncated 2B. Consistent with this, no product of the expected size of 2A-2B (∼30 kDa) was observed in the radiolabeling experiment using LVWT virus. Instead, a product (or products) migrating slightly more slowly than WT 2A was observed, consistent with either 2A-2B* or 2A-2B^N^. Mass-spectrometric analysis of V5-tagged LVWT confirmed that V5-tagged products migrating at this size comprise both V5-tagged 2A-2B* and V5-tagged 2A-2B^N^. If N-terminally truncated 2B can carry out all necessary 2B functions, then it would appear that the importance of StopGo is to produce the correct C terminus of 2A and/or the correct N terminus of 2B*. The differing susceptibilities of EMCV and TMEV to StopGo inhibition may be due to 2B* being functional in EMCV but not in TMEV, or perhaps due to the 2B* that is appended to 2A having a greater inhibitory effect on 2A function or L-1ABCD-2A2B* polyprotein processing in EMCV due to the larger size of EMCV 2B* (cf. the polyprotein processing studies in reference 8).

Frameshifting downregulates production of the TMEV nonstructural proteins 2BC-3ABCD by 74 to 82%. During the course of viral infection, the structural proteins are required in much greater quantities than the enzymatic proteins, in particular the viral polymerase. In many RNA viruses, transcriptional or translational control mechanisms are used to downregulate production of the latter relative to the former (reviewed in reference 28). However, in viruses that use a single-polyprotein expression strategy, such mechanisms would appear to be unavailable, and any regulation of relative protein levels must normally occur posttranslationally, e.g., via polyprotein processing, protein turnover, the production of inactive conformers, or sequestration of enzymatic proteins in inclusion bodies or the nucleus. We suggest that highly efficient −1 PRF in TMEV provides a mechanism to escape the confines of a single-polyprotein expression strategy, allowing efficient downregulation of replication protein synthesis while simultaneously releasing translational resources for enhanced structural protein synthesis.

## Supplementary Material

Supplemental material

## References

[1] Palmenberg AC, Kirby EM, Janda MR, Drake NL, Duke GM, Potratz KF, Collett MS 1984 The nucleotide and deduced amino acid sequences of the encephalomyocarditis viral polyprotein coding region. Nucleic Acids Res 12:2969–2985. doi:10.1093/nar/12.6.2969. 6324136 PMC318719

[2] Jackson RJ 1986 A detailed kinetic analysis of the in vitro synthesis and processing of encephalomyocarditis virus products. Virology 149:114–127. doi:10.1016/0042-6822(86)90092-9. 3004023

[3] Palmenberg AC 1990 Proteolytic processing of picornaviral polyprotein. Annu Rev Microbiol 44:603–623. doi:10.1146/annurev.mi.44.100190.003131. 2252396

[4] Batson S, Rundell K 1991 Proteolysis at the 2A/2B junction in Theiler's murine encephalomyelitis virus. Virology 181:764–767. doi:10.1016/0042-6822(91)90914-W. 2014649

[5] Palmenberg AC, Parks GD, Hall DJ, Ingraham RH, Seng TW, Pallai PV 1992 Proteolytic processing of the cardioviral P2 region: primary 2A/2B cleavage in clone-derived precursors. Virology 190:754–762. doi:10.1016/0042-6822(92)90913-A. 1325705

[6] Atkins JF, Wills NM, Loughran G, Wu C-Y, Parsawar K, Ryan MD, Wang C-H, Nelson CC 2007 A case for “StopGo”: reprogramming translation to augment codon meaning of GGN by promoting unconventional termination (Stop) after addition of glycine and then allowing continued translation (Go). RNA 13:803–810. doi:10.1261/rna.487907. 17456564 PMC1869043

[7] Ryan MD, Drew J 1994 Foot-and-mouth disease virus 2A oligopeptide mediated cleavage of an artificial polyprotein. EMBO J 13:928–933. 8112307 10.1002/j.1460-2075.1994.tb06337.x PMC394894

[8] Hahn H, Palmenberg AC 1996 Mutational analysis of the encephalomyocarditis virus primary cleavage. J Virol 70:6870–6875. 8794329 10.1128/jvi.70.10.6870-6875.1996 PMC190735

[9] Donnelly ML, Gani D, Flint M, Monaghan S, Ryan MD 1997 The cleavage activities of aphthovirus and cardiovirus 2A proteins. J Gen Virol 78:13–21. 9010280 10.1099/0022-1317-78-1-13

[10] Hahn H, Palmenberg AC 2001 Deletion mapping of the encephalomyocarditis virus primary cleavage site. J Virol 75:7215–7218. doi:10.1128/JVI.75.15.7215-7218.2001. 11435606 PMC114454

[11] Doronina VA, Wu C, de Felipe P, Sachs MS, Ryan MD, Brown JD 2008 Site-specific release of nascent chains from ribosomes at a sense codon. Mol Cell Biol 28:4227–4239. doi:10.1128/MCB.00421-08. 18458056 PMC2447138

[12] Brierley I, Jenner AJ, Inglis SC 1992 Mutational analysis of the “slippery-sequence” component of a coronavirus ribosomal frameshifting signal. J Mol Biol 227:463–479. doi:10.1016/0022-2836(92)90901-U. 1404364 PMC7125858

[13] Brierley I, Gilbert R, Pennell S 2010 Pseudoknot-dependent programmed −1 ribosomal frameshifting: structures, mechanisms and models, p 149–174. *In* Atkins J, Gesteland R (ed), Recoding: expansion of decoding rules enriches gene expression. Springer, Heidelberg, Germany.

[14] Miller W, Giedroc D 2010 Ribosomal frameshifting in decoding plant viral RNAs, p 193–220. *In* Atkins J, Gesteland R (ed), Recoding: expansion of decoding rules enriches gene expression. Springer, Heidelberg, Germany.

[15] Takyar S, Hickerson RP, Noller HF 2005 mRNA helicase activity of the ribosome. Cell 120:49–58. doi:10.1016/j.cell.2004.11.042. 15652481

[16] Qu X, Wen J-D, Lancaster L, Noller HF, Bustamante C, Tinoco I 2011 The ribosome uses two active mechanisms to unwind messenger RNA during translation. Nature 475:118–121. doi:10.1038/nature10126. 21734708 PMC4170678

[17] Plant EP, Dinman JD 2005 Torsional restraint: a new twist on frameshifting pseudoknots. Nucleic Acids Res 33:1825–1833. doi:10.1093/nar/gki329. 15800212 PMC1072802

[18] Namy O, Moran SJ, Stuart DI, Gilbert RJC, Brierley I 2006 A mechanical explanation of RNA pseudoknot function in programmed ribosomal frameshifting. Nature 441:244–247. doi:10.1038/nature04735. 16688178 PMC7094908

[19] Loughran G, Firth AE, Atkins JF 2011 Ribosomal frameshifting into an overlapping gene in the 2B-encoding region of the cardiovirus genome. Proc Natl Acad Sci U S A 108:E1111–E1119. doi:10.1073/pnas.1102932108. 22025686 PMC3219106

[20] Law KM, Brown TD 1990 The complete nucleotide sequence of the GDVII strain of Theiler's murine encephalomyelitis virus (TMEV). Nucleic Acids Res 18:6707–6708. doi:10.1093/nar/18.22.6707. 2251141 PMC332652

[21] Sorgeloos F, Jha BK, Silverman RH, Michiels T 2013 Evasion of antiviral innate immunity by Theiler's virus L* protein through direct inhibition of RNase L. PLoS Pathog 9:e1003474. doi:10.1371/journal.ppat.1003474. 23825954 PMC3694852

[22] Loughran G, Libbey JE, Uddowla S, Scallan MF, Ryan MD, Fujinami RS, Rieder E, Atkins JF 2013 Theiler's murine encephalomyelitis virus contrasts with encephalomyocarditis and foot-and-mouth disease viruses in its functional utilization of the StopGo non-standard translation mechanism. J Gen Virol 94:348–353. doi:10.1099/vir.0.047571-0. 23100365 PMC3709618

[23] Yan S, Wen J-D, Bustamante C, Tinoco I 2015 Ribosome excursions during mRNA translocation mediate broad branching of frameshift pathways. Cell 160:870–881. doi:10.1016/j.cell.2015.02.003. 25703095 PMC4344849

[24] Grentzmann G, Ingram JA, Kelly PJ, Gesteland RF, Atkins JF 1998 A dual-luciferase reporter system for studying recoding signals. RNA 4:479–486. 9630253 PMC1369633

[25] Fixsen SM, Howard MT 2010 Processive selenocysteine incorporation during synthesis of eukaryotic selenoproteins. J Mol Biol 399:385–396. doi:10.1016/j.jmb.2010.04.033. 20417644 PMC2916059

[26] Girnary R, King L, Robinson L, Elston R, Brierley I 2007 Structure-function analysis of the ribosomal frameshifting signal of two human immunodeficiency virus type 1 isolates with increased resistance to viral protease inhibitors. J Gen Virol 88:226–235. doi:10.1099/vir.0.82064-0. 17170455

[27] Li Y, Treffers EE, Napthine S, Tas A, Zhu L, Sun Z, Bell S, Mark BL, van Veelen PA, van Hemert MJ, Firth AE, Brierley I, Snijder EJ, Fang Y 2014 Transactivation of programmed ribosomal frameshifting by a viral protein. Proc Natl Acad Sci U S A 111:E2172–E2181. doi:10.1073/pnas.1321930111. 24825891 PMC4040542

[28] Ahlquist P 2006 Parallels among positive-strand RNA viruses, reverse-transcribing viruses and double-stranded RNA viruses. Nat Rev Microbiol 4:371–382. doi:10.1038/nrmicro1389. 16582931 PMC7097367

