# Supplementary material for: Characterization of Ribosomal Frameshifting in Theiler's Murine Encephalomyelitis Virus
Source: J Virol. 2015 Jun 10;89(16):8580–9. doi: 10.1128/JVI.01043-15 (PMC4524249; doi:10.1128/JVI.01043-15)
Supplement: Supplemental material [file supp_89_16_8580__index.html]

Characterization of Ribosomal Frameshifting in Theiler's Murine Encephalomyelitis Virus — Supplemental material 

# Characterization of Ribosomal Frameshifting in Theiler's Murine Encephalomyelitis Virus

## Supplemental material

- Supplemental file 1 -

  Fig. S1 (Mass spectrometric analysis of tagged products.)

  PDF, 1.2M
