# Supplementary material for: Characterization of Ribosomal Frameshifting in Theiler's Murine Encephalomyelitis Virus
Source: J Virol. 2015 Jun 10;89(16):8580–9. doi: 10.1128/JVI.01043-15 (PMC4524249; doi:10.1128/JVI.01043-15)

Supplementary Figure S1. Mass spectrometric analysis of tagged products. (A) Fragmentation spectrum of the shift-site peptide derived from V5-tagged 2A-2B*. **(B)** Fragmentation spectrum of a peptide consistent with 3C-Pro cleavage of tagged 2A-2B at the conserved Q|G encoded just downstream of the frameshift site. **(C)** Fragmentation spectrum of the shift-site peptide derived from HA-tagged 2A-2B*.

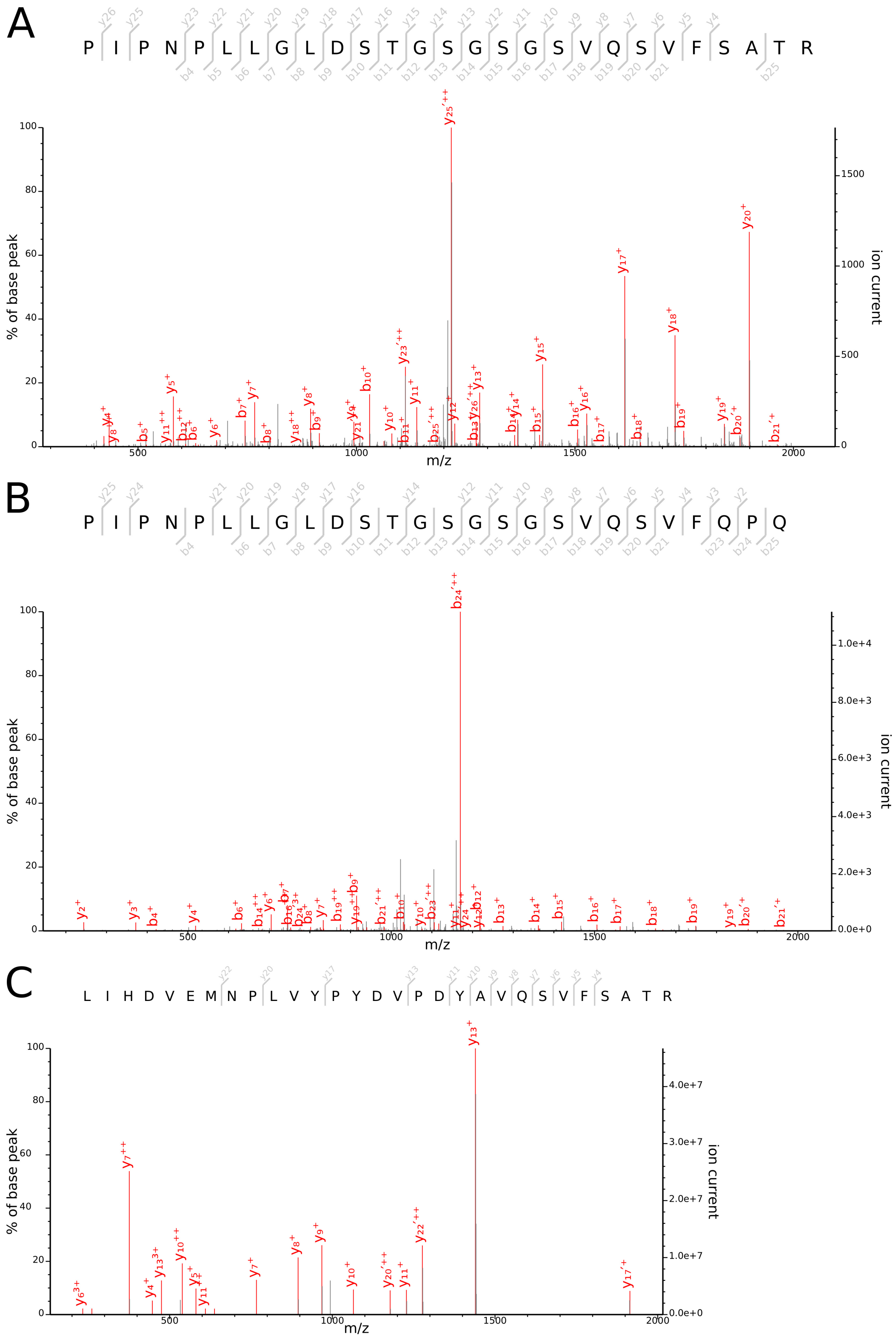

Supplement: Supplemental material [file JVI.01043-15_zjv999090677so1.pdf]
